# Bevacizumab Efficiently Inhibits VEGF-Associated Cellular Processes in Equine Umbilical Vein Endothelial Cells: An In Vitro Characterization

**DOI:** 10.3390/vetsci10110632

**Published:** 2023-10-26

**Authors:** Ulrike Lessiak, Barbara Pratscher, Alexander Tichy, Barbara Nell

**Affiliations:** 1 Department of Companion Animals and Horses, University of Veterinary Medicine Vienna, Veterinaerplatz 1, 1210 Vienna, Austria; 2 Department of Biomedical Sciences, University of Veterinary Medicine Vienna, Veterinaerplatz 1, 1210 Vienna, Austria

**Keywords:** anti-VEGF, equine umbilical vein endothelial cells, bevacizumab, angiogenesis

## Abstract

**Simple Summary:**

Angiogenesis, the sprouting of new capillaries from existing vessels, plays a crucial role in various physiological processes. Pathological vascularization, as seen in, e.g., cancer, musculoskeletal diseases, or ocular disorders, eventually causes tissue and organ dysfunctions. Vascular endothelial growth factor A (VEGF-A) and its receptors are known to play a key role in angiogenesis; therefore, targeting the VEGF pathway represents one of the main areas of human cancer research in recent years. So far, anti-VEGF treatment in horses has not been investigated. In this study, the effect of bevacizumab, the most widespread anti-VEGF agent, on an equine cell line harvested from umbilical cords was investigated. Bevacizumab efficiently inhibited various cellular processes associated with angiogenesis and could therefore be a promising therapeutic approach in vascular-driven diseases in horses.

**Abstract:**

Anti-VEGF agents were found to have clinical implications for the successful treatment of vascular-driven diseases in humans. In this study, a detailed biological characterization of bevacizumab in a variety of in vitro assays was carried out to determine the effect of bevacizumab on equine umbilical vein endothelial cells (EqUVEC). EqUVECs were harvested from umbilical cords of clinically healthy horses and exposed to different concentrations (1, 2, 4, 6, 8 mg/mL) of bevacizumab (Avastin^®^). Assays concerning the drug’s safety (cell viability and proliferation assay) and efficacy (cell tube formation assay, cell migration assay, and Vascular endothelial growth factor (VEGF) expression) were carried out reflecting multiple cellular processes. Bevacizumab significantly decreased VEGF expression at all concentrations over a 72 h period. No cytotoxic effect of bevacizumab on EqUVECs was observed at concentrations of 4 mg/mL bevacizumab or lower. Incubated endothelial cells showed delayed tube formation and bevacizumab efficiently inhibited cell migration in a dose-dependent manner. Bevacizumab potently inhibits VEGF-induced cellular processes and could be a promising therapeutic approach in vascular-driven diseases in horses.

## 1. Introduction

Angiogenesis plays a critical role in repairing, expanding, and remodeling tissues [1]. In healthy organisms, a balance of pro- and anti-angiogenic factors tightly regulates the sprouting of new capillaries from existing vessels [2]. Multiple signaling molecules are involved in the process, which are often overlapping. They lead to the proliferation and migration of endothelial cells, vascular tube formation, and the establishment of vascular networks. An imbalanced expression of angiogenic factors may lead to pathological vascularization, eventually causing tissue and organ dysfunctions [3,4]. Given the diverse activities of the vasculature, targeting angiogenesis is likely to be a suitable approach for addressing many diseases. 

VEGF-A and its receptors are known to play a key role in angiogenesis, as shown in experimental models of corneal neovascularization (CNV) [5]. In 2004, bevacizumab (Avastin^®^, Roche, Basel, Switzerland), a recombinant humanized anti-VEGF immunoglobulin G1 (IgG 1) antibody, was invented. Originally approved by the FDA for the treatment of colorectal cancer, bevacizumab remains one of the most widely used anti-cancer drugs, with FDA approvals for various indications [6]. Additionally, it has shown off-label clinical applications in ophthalmology in the successful management of vascular-related diseases [7,8,9]. The VEGF pathway is now targeted by almost all clinically approved anti-angiogenic drugs for addressing cancer and ocular diseases [1,10]. 

Given the various biological functions of VEGF-A, anti-VEGF drugs are likely to act through complex processes such as anti-angiogenesis, tumor vessel regression, and normalization and reduction of vascular leakage. These processes help improve the effectiveness of anti-cancer drug delivery [11]. Additionally, animal models have demonstrated suppression of tumor growth and metastasis by neutralizing VEGF through prevention of tumor transformation (“angiogenic switch”), thus maintaining a quiescent state [12]. Furthermore, it contributes to the primitive vasculature development and other relevant biological processes in a variety of tumors [13]. Since VEGF ligand families are overexpressed in most solid cancers, standard therapeutics in human cancer therapy include inhibition of the VEGF–VEGFR2 axis by administering anti-VEGF antibodies [14].

In comparison, there is relatively little ongoing research on the importance of angiogenesis and pro- and anti-angiogenic treatments for horses [4]. Nevertheless, VEGF expression was found to correlate with pathologies as diverse as impaired wound healing, ocular disorders, musculoskeletal diseases, laminitis, and many forms of cancer [15,16,17,18,19,20,21,22]. Furthermore, bevacizumab (Avastin^®^, Roche), one of the most potent VEGF inhibitors, has already demonstrated its safety and effectiveness in the treatment of CNV in dogs [23].

However, anti-VEGF treatment in horses has not been investigated previously. To assess the anti-angiogenic effect of bevacizumab in vitro, equine umbilical vein endothelial cells (EqUVEC) were used as an equivalent to human umbilical vein endothelial cells (HUVEC). We hypothesized that bevacizumab inhibits VEGF-associated cellular processes in EqUVECs efficiently. A detailed biological characterization of bevacizumab on EqUVECs was carried out in a variety of in vitro models concerning the drug’s safety (cell viability and proliferation assay) and efficacy (cell tube formation assay, cell migration assay, and VEGF expression).

## 2. Materials and Methods

### 2.1. Drug Preparation

Bevacizumab 25 mg/mL (Avastin^®^) was obtained from Roche (Basel, Switzerland). Solutions of different concentrations (1, 2, 4, 6, and 8 mg/mL) of bevacizumab were aseptically prepared in compliance with good manufacturing practice with sterile 0.9% saline solution as a solvent. Cell culture medium was used as a control (Ctrl, 0 mg/mL bevacizumab). 

### 2.2. ELISA for EqUVEC VEGF Expression

The expression of VEGF was analyzed from EqUVEC cell culture supernatants collected at 0 h, 24 h, 48 h, and 72 h of incubation with 0, 1, 2, 4, 6, and 8 mg/mL bevacizumab. Concentrations of VEGF were evaluated using a commercially available, species-specific ELISA kit (Equine VEGF-A ELISA Kit, Invitrogen, Waltham, MA, USA). According to the manufacturer’s instructions, standards and samples were added to each well and incubated for 2.5 h at room temperature. The wells were incubated with biotinylated equine VEGF-A for 1 h at room temperature whilst being gently shaken. Streptavidin HRP was diluted 1:200 and added to each well. The peroxidase activity was determined by incubation with 100 μL peroxidase substrate solution 3,3′,5,5′-tetramethylbenzidine (TMB). Color development was stopped after 30 min. Absorbance at 450 nm was quantified in a microplate reader (Promega Corporation, Fitchburg, WI, USA). The standard curve was obtained by serial dilutions of VEGF-A. The linear range was between 1.95 pg/mL to 62.5 pg/mL. Experiments were carried out in triplicates. 

### 2.3. Cell Culture

Cells were isolated and cultured from umbilical cords of clinically healthy horses (manuscript in preparation). Owners provided informed written consent before hospitalization of the patient. Briefly, endothelial cells were isolated within 12 h after harvesting. After detachment, cells were grown in a high glucose basic culture medium (DMEM + GlutaMAX ™, Life Technologies, Thermofisher Science, Carlsbad, CA, USA) supplemented with 20% FBS, 15 mM HEPES (Life Technologies, Thermofisher Science, Carlsbad, CA, USA) and antibiotic–antimycotic 100X (Life Technologies, Thermofisher Science, Carlsbad, CA, USA) in a 37 °C and 5% CO_2_ humidified incubator. Cells were seeded at 5000 cells/cm^2^. After reaching confluence, the monolayer was washed with PBS and trypsinized (trypsin–EDTA for primary cells, ATCC^®^, Manassas, VA, USA) before being used in experiments. EqUVECs up to the fifth passage were used in all experiments.

### 2.4. EqUVEC Cell Viability 

To determine bevacizumab’s safety on EqUVECs the effect on cell viability was studied by measuring the cells’ potential to reduce the assay substrate and thus metabolism. Cell viability was assayed in situ at a 24 h interval for 72 h using the RealTime-Glo™ MT Cell Viability Assay (Promega Corporation, Fitchburg, WI, USA) according to the manufacturer’s protocol. Briefly, cells were inoculated at 1.6 × 10^4^ cells/well onto an opaque-walled 96-well plate (Falcon; Becton Dickinson Labware, Plymouth, UK). Upon confluence, cells were treated with different concentrations of bevacizumab and RealTime-Glo™, MT Cell Viability Substrate, and NanoLuc^®^ Enzyme were added. After 1 h of incubation at 37 °C in a 5% CO_2_ humidified chamber, luminescence was measured using the GloMax^®^ Explorer Multimode Microplate Reader (Promega Corporation, Fitchburg, WI, USA). Experiments were carried out in triplicates. 

### 2.5. EqUVEC Cell Proliferation

For determination of viable cells in proliferation an MTS assay (3-(4,5-dimethylthiazol-2-yl)-5-(3-carboxymethoxyphenyl)-2-(4-sulfophenyl)-2H-tetrazolium) was conducted using the CellTiter 96^®^ AQueous One Solution Cell Proliferation Assay (Promega Corporation, Fitchburg, WI, USA). Briefly, cells were plated (1.6 × 10^4^ cells/well) on 96-well plates and incubated with different concentrations of bevacizumab for 72 h. At the end of treatment, 20 µL/well of CellTiter 96AQueous One Solution (MTS) solution (Promega Corporation, Fitchburg, WI, USA) was added and incubated at 37 °C in a 5% CO_2_ humidified chamber for 1 h. Absorbance was measured at 490 nm using the GloMax^®^ Explorer Multimode Microplate Reader (Promega Corporation, Fitchburg, WI, USA). Experiments were carried out in triplicates.

### 2.6. EqUVEC Tube Formation

In order to determine the effect of bevacizumab on equine angiogenesis, tube formation assays were prepared as follows. Matrigel (10 μL, Thermofisher Science, Carlsbad, CA, USA) was added to each well of a chilled ibidi μ- angiogenesis slide (Ibidi GmbH, Munich, Germany). Polymerization of the Matrigel was achieved by incubation at 37 °C for 30 min. The cells were harvested and 2 × 10^5^ EqUVECs were seeded and treated with different concentrations of bevacizumab. Angiogenesis slides were allowed to incubate at 37 °C in 5% CO_2_ humidified ambient oxygen conditions for 30 min. Whole wells were imaged at 1–3 h intervals for up to 14 h using 10× phase contrast magnification on a Leica DMi8 inverted microscope (Leica Microsystems GmbH, Wetzlar, Germany) with fixed X and Y positions for each well. Number of tubes, number of junctions, and total length of branches and segments were quantified using the Angiogenesis Analyzer plugin (Supplementary Figure S1) for ImageJ/Fiji^®^ [24]. Experiments were carried out in duplicates in two different experiments. 

### 2.7. EqUVEC Cell Migration

EqUVECs (1 × 10^5^/well) were cultured onto a 12-well plate. At confluent conditions, a scratch was introduced with a sterile 1000 μL tip. Cell debris was removed by washing with PBS. Cells were treated with different concentrations of bevacizumab for 72 h. Scratch filling was documented using a Leica DMi8 inverted microscope (Leica Microsystems GmbH, Wetzlar, Germany) with fixed X and Y positions (4× magnification) for at least three positions for each well at 24 h intervals (0 h, 24 h, 48 h, and 72 h). Wounded area was quantified using the Wound Healing Size Tool plugin (Supplementary Figure S2) for ImageJ/Fiji^®^ [25]. Experiments were carried out in triplicates. 

### 2.8. Statistics

The results of the experiments performed in triplicates are expressed as mean values with 95% confidence interval. Multiple t-tests were conducted to assess if the treatment groups significantly differed from the control group at single time points. To counteract the problem of multiple comparisons the Holm method was used as alpha error correction procedure. VEGF levels, cell viability, and cell proliferation were expressed as a percentage of the measured absorbance of control cells. Tube formation assays were performed in different experimental set-ups and therefore the results were considered from only one single experiment and presented in a descriptive manner. Migration was analyzed expressing the wounded area in percentage of total area. *p*-values < 0.05 were considered significant. The analyses were performed with IBM^®^ SPSS^®^ 28.0 (IBM Corporation, New York, NY, USA) software and RStudio 9.3.191.223 (Posit PBC, Boston, MA, USA).

## 3. Results

### 3.1. Bevacizumab Significantly Decreased EqUVEC VEGF Tissue Expression 

**Figure 1:**
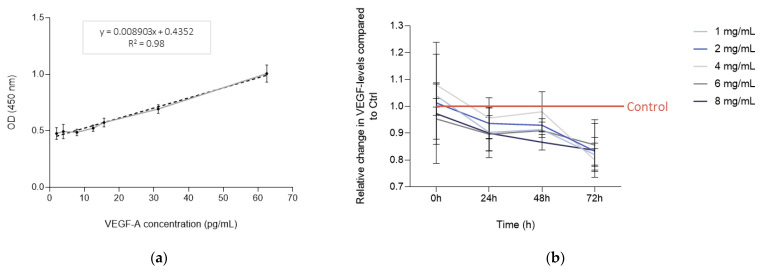
VEGF levels in the supernatants of the EqUVEC cell culture. (**a**) An ELISA standard curve was established to detect 1.95 pg/mL to 62.5 pg/mL of VEGF-A as a reference. The standard curve was generated (n = 3) and data points are presented as means with 95% CI. The dotted line represents the best fit determined by linear curve fitting (r^2^ = 0.98). OD = optical density. (**b**) Relative VEGF expression after 0, 24, 48, and 72 h of incubation with different concentration levels of bevacizumab (1, 2, 4, 6, and 8 mg/mL). Results are shown as means with 95% CI (n = 3).

The inhibitory effect of bevacizumab on VEGF levels in cell culture supernatants was investigated. A standard curve was established using a fresh vial of Avastin^®^ between 1.95 pg/mL and 62.5 pg/mL. The detected range resulted in a linear relationship (Figure 1a). Reduced VEGF expression was achieved in EqUVECs when treated with bevacizumab (1, 2, 4, 6, and 8 mg/mL) at all time points compared to control. Statistically significant inhibition was observed in cells exposed to 1 mg/mL (n = 3; 24 h: *p* = 0.03; 48 h: *p* < 0.01; 72 h: *p* = 0.02; Table 1) and 2 mg/mL bevacizumab (n = 3; 24 h: *p* = 0.05; 48 h: *p* = 0.03; 72 h: *p* = 0.01; Table 1). EqUVECs exposed to 4 mg/mL of bevacizumab showed likewise decreased VEGF expression in comparison to those exposed to medium alone, although not statistically significant after 24 h (n = 3; *p* = 0.11; Table 1) and 48 h (n = 3; *p* = 0.58; Table 1) and statistically significant after 72 h (n = 3; *p* = 0.02; Table 1). Equally, bevacizumab downregulated the expression of VEGF statistically significant when incubated with 6 mg/mL (n = 3; 24 h: *p* = 0.02; 48 h: *p* < 0.01; 72 h: *p* = 0.02; Table 1) and 8 mg/mL (n = 3; 24 h: *p* = 0.05; 48 h: *p* = 0.01; 72 h: *p* = 0.02; Table 1) over a 72 h period. Relative inhibition of VEGF expression in cell culture supernatants of EqUVECs treated with different concentrations of bevacizumab (1, 2, 4, 6, and 8 mg/mL) compared to the control group is shown in Table 2.

### 3.2. Bevacizumab Significantly Decreased EqUVEC Cell Viability at Higher Concentrations

**Figure 2:**
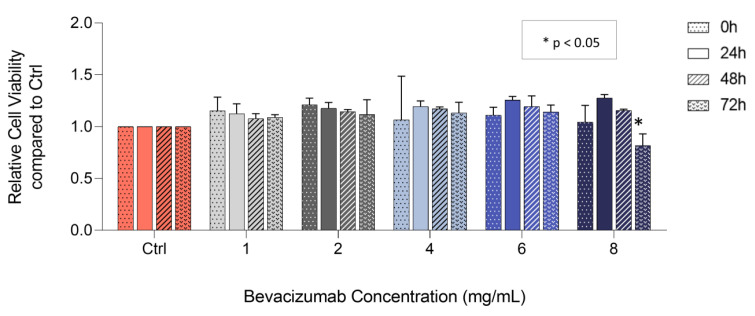
Viability assay after treatment with 1, 2, 4, 6, and 8 mg/mL bevacizumab. Results are shown as means (n = 3). Change in cell viability compared to control over a 72 h period (real-time assay). Statistically significant decrease in relative cell viability is shown as * (*p* < 0.05).

The inhibitory effect of bevacizumab on VEGF expression in cell culture supernatants led to further investigations regarding the effect of bevacizumab on endothelial cellular processes commonly associated with VEGF. Cytotoxicity assays were carried out in order to evaluate cell viability. When analyzed over a 72 h culture period, no decrease in relative cell viability was observed when treated with 1, 2, 4, and 6 mg/mL bevacizumab (n = 3; Figure 2; Table 3) in comparison to control throughout the time course. Similarly, cell viability of EqUVECs exposed to 8 mg/mL showed no decrease in cell viability when investigated at 24 h and 48 h (n = 3, Figure 2; Table 3). However, bevacizumab significantly decreased cell viability in EqUVECs after 72 h of treatment (n = 3, *p* = 0.02, Figure 2; Table 3) compared to control.

### 3.3. Bevacizumab Significantly Decreased EqUVEC Cell Proliferation

**Figure 3:**
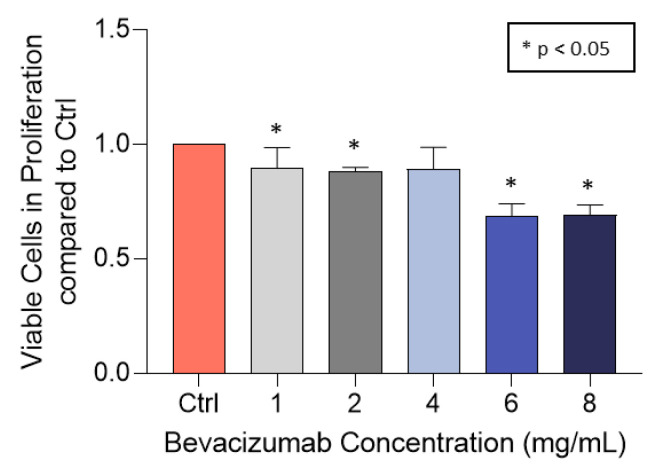
Proliferation assay after treatment with 1, 2, 4, 6, and 8 mg/mL bevacizumab. Results are shown as means (n = 3). Change in relative cell proliferation compared to control measured by MTS assay over a 72 h period (end-point assay). Statistically significant decrease in relative cell viability is shown as * (*p* < 0.05).

To determine viable cells in proliferation (end-point assay), EqUVECs were treated with increasing concentrations of bevacizumab (1, 2, 4, 6, and 8 mg/mL) or medium alone (Ctrl) for 72 h. Subsequently, the MTS tetrazolium compound was added in order to initiate a color change after reduction by metabolically active cells. A statistically significant decrease in cell proliferation was observed in cells treated with 1 mg/mL (n = 3, *p* = 0.04, Figure 3; Table 4), 2 mg/mL (n = 3, *p* < 0.01, Figure 3; Table 4) and 4 mg/mL bevacizumab (n = 3, *p* = 0.04, Figure 3; Table 4). Cells exposed to 6 mg/mL (n = 3, *p* < 0.01, Figure 3; Table 4) and 8 mg/mL (n = 3, *p* < 0.01, Figure 3; Table 4) bevacizumab showed a similar decrease although it resulted in a greater reduction of proliferative cells. Considering this, higher concentrations of bevacizumab (>6 mg/mL) were excluded from further investigation due to possible toxic effects.

### 3.4. Bevacizumab Inhibited EqUVEC Tube Formation

**Figure 4:**
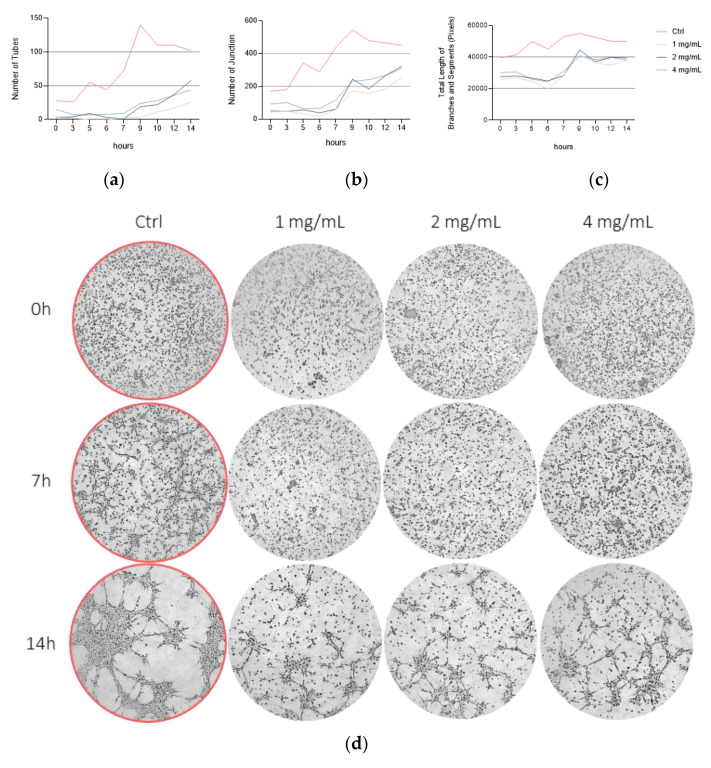
EqUVEC tubular network formation in in vitro angiogenesis assay. EqUVEC cells growing on Matrigel overnight in the presence of 1, 2, or 4 mg/mL bevacizumab or medium alone (Ctrl). The effects on polygon formation and tube length were visualized over a 14 h period. (**a**) Number of polygons corresponding to closed areas delimited by segments and associated junctions per well. (**b**) Number of junctions per well. (**c**) Total number of branches and segment length per well. (**d**) EqUVEC cells growing on Matrigel overnight in the presence of bevacizumab or medium alone (Ctrl). (**d**) Representative images of treated (1, 2, or 4 mg/mL bevacizumab) and untreated (Ctrl) EqUVECs after 0 h, 7 h, and 14 h of incubation.

In order to investigate the effect of bevacizumab in equine angiogenesis, descriptive tube formation assays were carried out. When analyzed over a 14 h culture period, bevacizumab inhibited the mean number of tubes in comparison to normal non-treated cells (Ctrl). These effects were consistent in all concentrations (1, 2, and 4 mg/mL bevacizumab) (n = 1; Ctrl: 28 (0 h)–102 (14 h), +7.63 tubes/h; 1 mg/mL: 3 (0 h)–26 (14 h), +1.65 tubes/h; 2 mg/mL: 3 (0 h)–58 (14 h), +3.75 tubes/h; 4 mg/mL: 15 (0 h)–44 (14 h), +2.60 tubes/h; Figure 4a). Similarly, cells exposed to bevacizumab had fewer junctions in comparison to those exposed to medium alone (n = 1; Ctrl: 170 (0 h)–452 (14 h), +25.68 junctions/h; 1 mg/mL: 43 (0 h)–252 (14 h), +16.69 junctions/h; 2 mg/mL: 50 (0 h)–323 (14 h), +22.53 junctions/h; 4 mg/mL: 94 (0 h)–309 (14 h), +18.77 junctions/h; Figure 4b). However, total length of segments and branches was only slightly decreased compared to control (n = 1; Ctrl: 39,537 (0 h)–49,888 (14 h); 1 mg/mL: 25,784 (0 h)–37,964 (14 h); 2 mg/mL: 27,553 (0 h)–39,462 (14 h); 4 mg/mL: 30,174 (0 h)–38,048 (14 h); Figure 4c). Representative images of treated (1, 2, or 4 mg/mL bevacizumab) and untreated (Ctrl) EqUVECs after 0 h, 7 h, and 14 h of incubation are shown in Figure 4d.

### 3.5. Bevacizumab Significantly Inhibited EqUVEC Cell Migration

**Figure 5:**
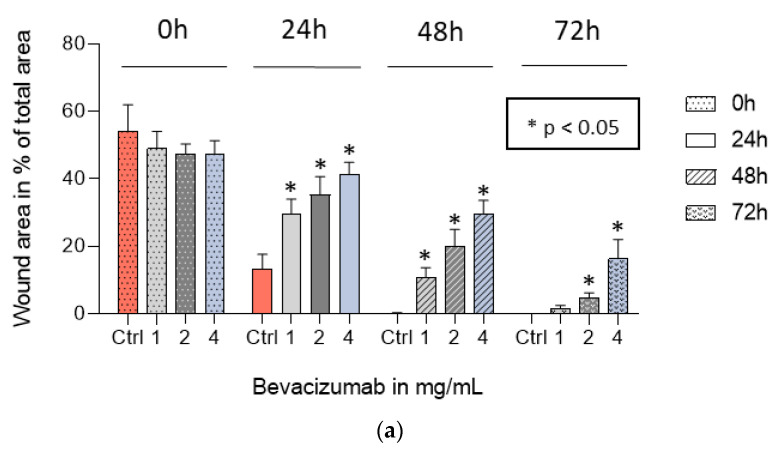

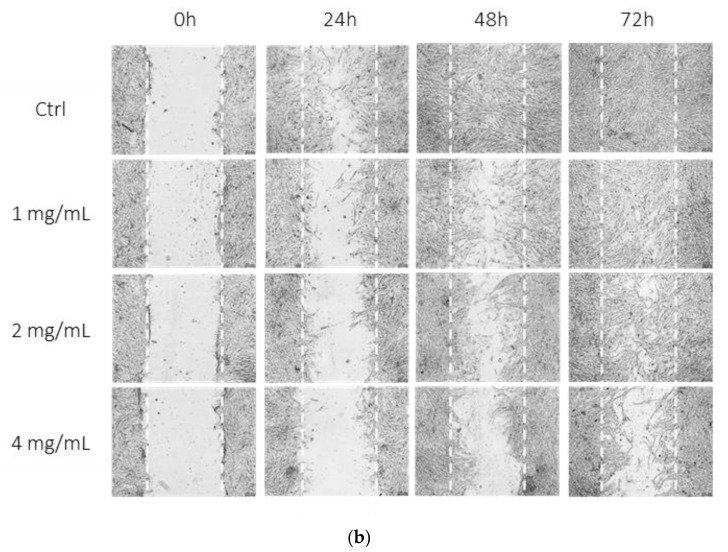
Migration assay after treatment with 1, 2, and 4 mg/mL of bevacizumab and without treatment (Ctrl). Results are shown as means (n = 9). (**a**) Percentage of wounded area at 24 h, 48 h, and 72 h post scratch is shown. Statistically significant decrease in relative cell migration is shown as * (*p* < 0.05). (**b**) Representative images after scratch wound. Images were taken over a 72 h period.

We assessed the efficacy of bevacizumab by comparing its capacity to reduce EqUVEC cell migration after scratch wound induction. Untreated cells (Ctrl) refilled the gap within 48 h (n = 9, Figure 5, Table 5). Incubation with 1 mg/mL bevacizumab statistically significantly inhibited cell migration for 48 h compared to control (n = 9; 24 h: *p* < 0.01; 48 h: *p* < 0.01; 72 h: *p* < 0.01; Figure 5, Table 5). Similarly, EqUVECs exposed to 2 mg/mL and 4 mg/mL showed a statistically significant delay in migration after 24 h (n = 9; 2 mg/mL: *p* < 0.01; 4 mg/mL: *p* < 0.01; Figure 5, Table 5) and 48 h (n = 9; 2 mg/mL: *p* < 0.01; 4 mg/mL: *p* < 0.01; Figure 5, Table 5). These cells never reached confluence and the effect lasted even after 72 h after wound initiation (n = 9; 2 mg/mL: *p* < 0.01; 4 mg/mL: *p* < 0.01; Figure 5, Table 5).

## 4. Discussion

In this study, the anti-angiogenic potential of bevacizumab on VEGF-associated cellular processes in equine umbilical vein endothelial cells (EqUVEC) was proven for the first time.

Angiogenesis plays a pivotal role in vascular-driven diseases and cancer development and metastasis representing one of the main areas of cancer research [11,26,27]. The VEGF–VEGFR signaling pathway is, among various others, the most studied and most important in tumor angiogenesis [28]. Topical and subconjunctival anti-VEGF agents have been used in a few studies for primary or recurrent ocular surface squamous neoplasia (OSSN) in humans, yielding mostly favorable clinical results without any ocular or systemic side effects [29,30,31,32,33]. In horses, squamous cell carcinoma (SCC) is the most prevalent tumor of the eye and its surrounding structures with a potential to metastasize [34,35,36]. It severely impacts the prognosis for vision and the long-term survival of affected animals. Following surgical removal alone, recurrence rates as high as 62% have been reported [34]. Consequently, adjunctive therapies may be necessary to prevent recurrences and manage advanced diseases. One possible approach might be the off-label use of anti-vascular endothelial growth factor (anti-VEGF) agents. Therefore, understanding the influence of bevacizumab on VEGF-associated cellular processes in equines is essential for future practical applications.

The VEGF receptor binding region between Arg 82 and Gly 91 amino acids was shown to be identical in equines and humans [37]. Thus, the binding activity of bevacizumab to equine VEGF can be assumed, although the interaction between bevacizumab and equine VEGF was not detected in vitro in an ELISA setup (Equine VEGF-A VetSet ELISA Development, Kingfisher, St. Paul, MN, USA) in a previous study [37]. To investigate the effect of bevacizumab on VEGF expression in equine EC, a different ELISA approach was chosen. An almost constant VEGF level could be maintained in cells treated with bevacizumab, whereas control cells upregulated their VEGF expression statistically significant by ~33%. On the contrary, Sadick et al. reported even a decline in VEGF levels in HUVECs when treated with 2, 4, 6, and 10 mg/mL bevacizumab [38]. In addition, VEGF expression in untreated cells was increased by 64%, indicating limited comparability between human and equine VEGF expression in endothelial cells and the need for further investigations.

Inhibition of VEGF expression led to further focus on VEGF-associated cellular processes in EqUVECs. Considering the safety and efficacy of bevacizumab, a diversity of in vitro assays such as viability, proliferation, tube formation, and migration assays and ELISA quantification of VEGF levels in cell culture supernatants of EqUVECS were performed. Cell viability was carried out with the aim of identifying safe dosages in equines. Cell viability was unaffected in cells treated with 6 mg/mL bevacizumab or lower over a period of 72 h. Incubation with higher dosages (8 mg/mL) led to a statistically significant decrease in cell viability after 72 h (82% viability compared to control). Similarly, bevacizumab affected proliferation in EqUVECs. The higher the concentration level of bevacizumab, the greater the decrease in proliferation with 88–89% in lower concentrations (1, 2, and 4 mg/mL bevacizumab) and 68% and 69% in cells treated with 6 mg/mL and 8 mg/mL bevacizumab, respectively. Given the direct correlation between the quantity of formazan product, assessed via absorbance at 490 nm, and the presence of viable cells within the culture, it was hypothesized that the cytotoxic effect of the drug may have led to endogenous apoptosis [39]. As described in the literature, this could potentially explain the observed decrease in cell proliferation, as opposed to a direct inhibition of cell proliferation itself [38,40]. Although topical bevacizumab is generally considered well-tolerated in human and veterinary ophthalmology [23,29,41], time and dose-dependent ocular toxicity, including mainly inhibitory effects on corneal wound healing, has been reported [42]. Although no side effects were observed in human patients with corneal neovascular diseases treated with 5 mg/mL topical bevacizumab for up to 12 months, the results considering the safety of usage in this study suggest that the risk of side effects may be reduced by using dosages as low as 4 mg/mL of bevacizumab in equines [29]. Apoptosis evaluation should have been conducted in order to establish safe concentrations for usage in horses.

Different methods were chosen to determine the anti-angiogenic effect of bevacizumab on EqUVECs. It revealed that bevacizumab efficiently inhibits EqUVEC tube formation and migration even when incubated with concentrations as low as 1 mg/mL. In HUVECs, bevacizumab likewise leads to an inhibition of tube formation and migration, although different concentrations and conditions were investigated [43,44].

Tube formation is a powerful tool to analyze vascular network formation in endothelial cells, reflecting the interplay of multiple cellular processes such as proliferation, migration, and apoptosis [43]. However, care must be taken when interpreting the tube formation assay given the use of a single replicate. Applying statistical analysis from multiple replicates would have helped to determine the significance of observed trends and differences, providing a more rigorous assessment of the experimental outcomes. Nevertheless, a clear trend could be shown when considering the slopes in the linear regression.

The limitations of the study are largely based on its in vitro character. Due to VEGF acting as a growth factor and promoting cell proliferation of HUVEC, those cells are primarily utilized in human angiogenesis research [38]. The presence of bevacizumab in the culture medium leads to an interaction between VEGF and bevacizumab, resulting in an inhibitory effect on HUVEC growth. Currently, there is no equine umbilical vein endothelial cell line commercially available; thus, a simple protocol for endothelial cell extraction from umbilical cords of mares was extrapolated from a HUVEC isolation protocol by Baudin et al. [45]. However, in vitro conditions are capable of mimicking in vivo conditions with limitations, e.g., endothelial cells (EC) are inhomogeneous, which could cause differences in angiogenic activity, and laboratory conditions disregard physiological forces [27]. To overcome cellular heterogeneity standardized preparations, media and passages were used. A combination of assays targeting different stages of angiogenesis was carried out to acquire compelling amounts of information regarding the process. Nonetheless, in vivo studies are needed to indicate the use of bevacizumab in patients with vascular-driven diseases.

## 5. Conclusions

This study shows that bevacizumab efficiently inhibits VEGF-associated cellular processes as seen in pathological angiogenesis, and thus may be taken into consideration as a possible therapeutic approach in equine medicine.

## Figures and Tables

**Table 1:** Absolute VEGF levels in pg/mL measured by ELISA in cell culture supernatants (n = 3).

	0 h				24 h				48 h				72 h			
Bevacizumab Concentration	VEGF (pg/mL)	95% CI ^1^	IQR ^2^	*p*-Value	VEGF (pg/mL)	95% CI ^1^	IQR ^2^	*p*- Value	VEGF (pg/mL)	95% CI ^1^	IQR ^2^	*p*- Value	VEGF (pg/mL)	95% CI ^1^	IQR ^2^	*p*-Value
Control	7.01	6.86–7.16	0.13		7.81	7.71–7.91	0.09		8.34	8.22–8.46	0.10		9.32	9.03–9.61	0.23	
1 mg/mL	7.30	7.14–7.46	0.12	0.19	7.05	6.68–7.42	0.30	0.03 *	7.60	7.48–7.72	0.09	<0.01 *	7.63	7.43–7.83	0.16	0.02 *
2 mg/mL	7.09	6.36–7.82	0.64	0.85	7.33	7.14–7.52	0.16	0.05 *	7.75	7.66–7.84	0.08	0.03 *	7.78	7.52–8.04	0.22	0.01 *
4 mg/mL	7.56	7.19–7.93	0.32	0.15	7.49	7.22–7.94	0.23	0.11	8.20	7.90–8.50	0.27	0.58	7.47	7.27–7.67	0.16	0.02 *
6 mg/mL	6.67	6.40–6.94	0.24	0.12	7.02	6.82–7.22	0.18	0.02 *	7.58	7.56–7.60	0.02	<0.01 *	7.99	7.61–8.37	0.30	0.02 *
8 mg/mL	6.80	6.44–7.17	0.28	0.30	7.05	6.80–7.30	0.21	0.05 *	7.23	7.11–7.36	0.11	0.01 *	7.81	7.43–8.19	0.34	0.02 *

* Statistically significant difference in absolute VEGF concentrations of treatment groups compared to VEGF concentrations measured in control group (*p* < 0.05). ^1^ CI = confidence interval. ^2^ IQR = interquartile range.

**Table 2:** Relative VEGF levels measured by ELISA in cell culture supernatants (n = 3).

	24 h				48 h				72 h			
Bevacizumab Concentration	Relative VEGF Level	95% CI ^1^	IQR ^2^	*p*-Value	Relative VEGF Level	95% CI ^1^	IQR ^2^	*p*-Value	Relative VEGF Level	95% CI ^1^	IQR ^2^	*p*-Value
Control	1.00				1.00				1.00			
1 mg/mL	0.90	0.85–0.95	0.04	0.06	0.91	0.89–0.93	0.01	0.01 *	0.82	0.79–0.85	0.02	<0.01 *
2 mg/mL	0.94	0.91–0.97	0.02	0.04 *	0.93	0.92–0.94	0.01	0.01 *	0.83	0.80–0.87	0.03	0.01 *
4 mg/mL	0.96	0.92–0.99	0.03	0.15	0.98	0.94–1.02	0.04	0.46	0.80	0.77–0.83	0.02	<0.01 *
6 mg/mL	0.90	0.87–0.93	0.03	0.02 *	0.91	0.90–0.91	<0.01	<0.01 *	0.86	0.80–0.91	0.05	0.02 *
8 mg/mL	0.90	0.87–0.94	0.03	0.03 *	0.87	0.85–0.89	0.02	<0.01 *	0.84	0.78–0.89	0.05	0.02 *

* Statistically significant difference in relative VEGF concentrations of treatment groups compared to VEGF concentrations measured in control group (*p* < 0.05). ^1^ CI = confidence interval. ^2^ IQR = interquartile range.

**Table 3:** Relative cell viability (n = 3).

	24 h			48 h			72 h		
Bevacizumab Concentration	Relative VEGF Level	95% CI ^1^	IQR ^2^	Relative VEGF Level	95% CI ^1^	IQR ^2^	Relative VEGF Level	95% CI ^1^	IQR ^2^
Control	1.00			1.00			1.00		
1 mg/mL	1.13	1.08–1.17	0.04	1.08	1.06–1.10	0.02	1.09	1.08–1.10	0.01
2 mg/mL	1.18	1.15–1.20	0.02	1.14	1.13–1.15	0.01	1.12	1.06–1.18	0.05
4 mg/mL	1.20	1.17–1.22	0.02	1.17	1.16–1.18	0.01	1.13	1.09–1.18	0.04
6 mg/mL	1.26	1.24–1.27	0.01	1.19	1.15–1.24	0.04	1.14	1.11–1.17	0.03
8 mg/mL	1.28	1.26–1.29	0.01	1.16	1.15–1.16	0.00	0.82 *	0.77–0.87	0.04

* Statistically significant reduction in relative cell viability of treatment group compared to control group (*p* = 0.02). ^1^ CI = confidence interval. ^2^ IQR = interquartile range.

**Table 4:** Relative cell proliferation (n = 3).

	72 h			
Bevacizumab Concentration	Relative VEGF Level	95% CI ^1^	IQR ^2^	*p*-Value
Control	1.00			
1 mg/mL	0.89	0.85–0.94	0.04	0.04 *
2 mg/mL	0.88	0.87–0.89	0.01	<0.01 *
4 mg/mL	0.89	0.85–0.94	0.04	0.04 *
6 mg/mL	0.68	0.66–0.71	0.02	<0.01 *
8 mg/mL	0.69	0.67–0.71	0.02	<0.01 *

* Statistically significant decrease in relative cell proliferation of treatment groups compared to control group (*p* < 0.05). ^1^ CI = confidence interval. ^2^ IQR = interquartile range.

**Table 5:** Percentage of wounded area at 24 h, 48 h, and 72 h after scratch (n = 9).

	0 h				24 h				48 h				72 h			
Bevacizumab Concentration	Area (%)	95% CI ^1^	IQR ^2^	*p*-Value	Area (%)	95% CI ^1^	IQR ^2^	*p*-Value	Area (%)	95% CI ^1^	IQR ^2^	*p*-Value	Area (%)	5% CI ^1^	IQR ^2^	*p*-Value
Control	54.20	47.44–60.96	7.12		13.23	10.51–15.95	3.85		0.13	0.05–0.21	5.56		0.00	-	-	
1 mg/mL	49.00	44.91–53.08	2.35	0.30	29.74	27.09–32.4	7.49	<0.01 *	10.57	8.99–12.15	3.21	<0.01 *	1.49	0.95–2.02	0.80	<0.01 *
2 mg/mL	47.35	44.97–49.74	3.84	0.17	35.27	31.94–38.61	5.32	<0.01 *	19.93	17.05–22.8	8.41	<0.01 *	4.83	3.97–5.69	0.87	<0.01 *
4 mg/mL	47.51	44.42–50.60	6.35	0.20	41.40	39.23–43.56	3.85	<0.01 *	29.64	27.28–32.00	4.58	<0.01 *	16.30	13.07–19.53	7.75	<0.01 *

* Statistically significant difference in wound area of treatment groups compared to control group (*p* < 0.05). ^1^ CI = confidence interval. ^2^ IQR = interquartile range.

## Data Availability

The datasets used and analyzed during the current study are available from the corresponding author on request.

## Supplementary Materials

The following supporting information can be downloaded at: https://www.mdpi.com/article/10.3390/vetsci10110632/s1, Figure S1: Tube formation assay analysis; Figure S2: Cell proliferation assay analysis.
