# Supplementary material for: Bevacizumab Efficiently Inhibits VEGF-Associated Cellular Processes in Equine Umbilical Vein Endothelial Cells: An In Vitro Characterization
Source: Vet Sci. 2023 Oct 26;10(11):632. doi: 10.3390/vetsci10110632 (PMC10675369; doi:10.3390/vetsci10110632)
Supplement: Supplementary file 1 [file vetsci-10-00632-s001.zip › vetsci-2613417-Supplementary Materials.pdf]

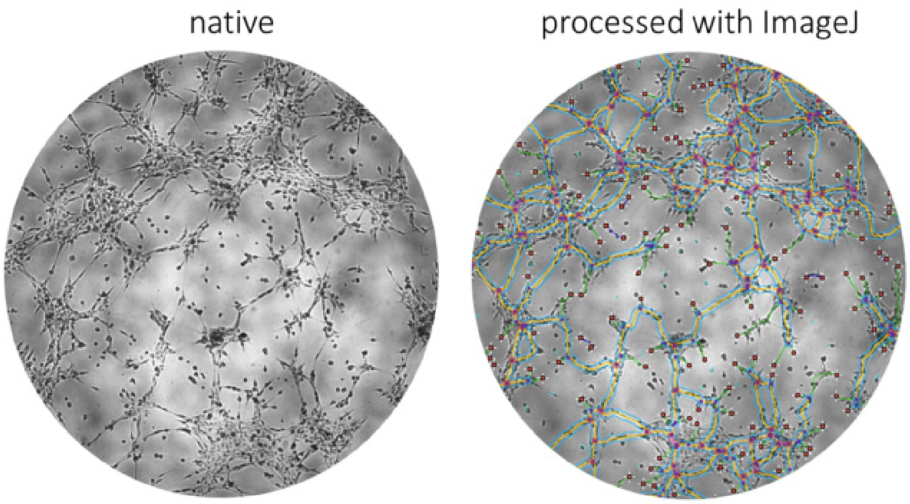

Figure S1: Tube formation assay analysis. Representative images (Ctrl, 10h) processed with ImageJ/Fiji® using the Angiongenesis Analyzer plugin.

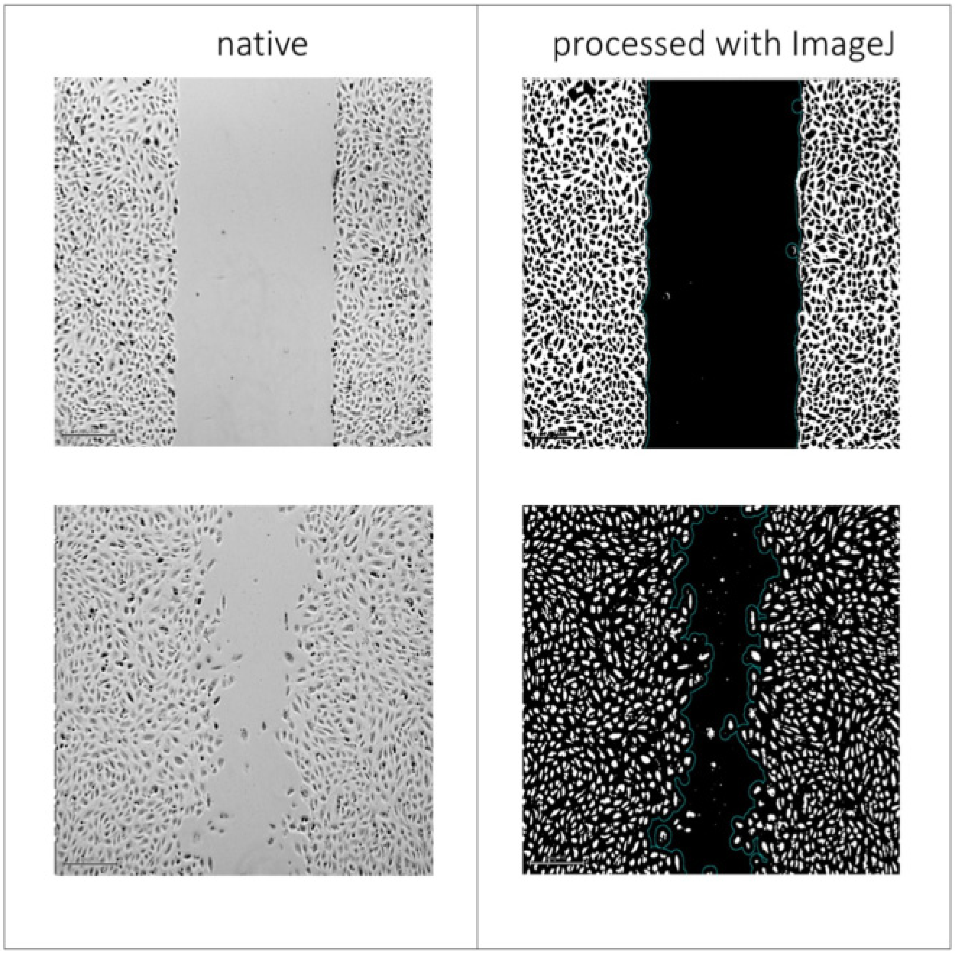

Figure S2: Cell proliferation assay analysis. Representative imags (Ctrl, 0h and 24h) processed with ImageJ/Fiji® using the Wound Healing Size Tool plugin.
